# A Case of Pregnancy Complicated with Evans Syndrome with Sequential Development of Autoimmune Warm Antibody Hemolytic Anemia and Idiopathic Thrombocytopenic Purpura

**DOI:** 10.1155/2019/2093612

**Published:** 2019-01-14

**Authors:** Haruka Suzuki, Koji Yamanoi, Jumpei Ogura, Takahiro Hirayama, Koji Yasumoto, Shimpei Shitanaka, Yoshihide Inayama, Mie Sakai, Tsutomu Ohara, Koh Suginami

**Affiliations:** Department of Obstetrics and Gynecology, Toyooka Public Hospital, 1094, Tobera, Toyooka City, Hyogo 6680065, Japan

## Abstract

The simultaneous or sequential development of autoimmune hemolytic anemia (AIHA) and idiopathic thrombocytopenic purpura (ITP) is known as Evans syndrome. We experienced a case of Evans syndrome that developed AIHA during pregnancy and ITP long after delivery. The patient was a 35-year-old pregnant woman (gravida 2, para 1). A routine blood test at 28 weeks of gestation revealed moderate macrocytic anemia. Her haptoglobin level was markedly low, and a direct antiglobulin test (DAT) was positive. Based on these results, AIHA was considered. A healthy female newborn with bodyweight 3575 g was vaginally delivered uneventfully. After delivery, the DAT remained positive, but anemia did not develop. At 203 days after delivery, ITP was detected. Because AIHA and ITP developed sequentially, she was diagnosed with Evans syndrome. When AIHA occurs during pregnancy, long-term follow-up is needed because ITP can develop sequentially.

## 1. Introduction

In a pregnant woman, the hemoglobin concentration and hematocrit decrease as a result of marked plasma augmentation. Moderate or severe anemia may, however, cause fetal growth restriction or premature birth [1, 2]. Thus, it is important to identify and properly manage the cause of anemia. Hemolytic anemia is one cause of moderate or severe anemia. Autoimmune hemolytic anemia (AIHA) is a type of hemolytic anemia.

AIHA is characterized by the development of anti-erythrocyte autoantibodies and the destruction of erythrocytes, which causes moderate or severe anemia [3, 4]. In some AIHA cases, idiopathic thrombocytopenic purpura (ITP) can develop simultaneously or sequentially, which is known as Evans syndrome [5, 6]. Because Evans syndrome is a rare disease, little is known about the relationship between pregnancy and Evans syndrome.

Here we report a case of pregnancy complicated with Evans syndrome. In this case, AIHA developed during pregnancy and ITP developed long after delivery. This is the first report of a case of AIHA and ITP developing sequentially at very different times.

## 2. Case Presentation

A 35-year-old woman, impregnated via intracytoplasmic sperm injection (ICSI), visited our hospital at 9 weeks of gestation. She had a history of one pregnancy with a normal delivery. The patient also had a history of asthma and no history of blood cell transfusion or medication except for the use of the antibiotic cephem during ICSI to prevent infection. A blood test administered at her first visit revealed that she was D-antigen-positive and irregular antibody-negative and her hemoglobin concentration was 14.4 g/dl.

At 28 weeks of gestation, a blood test revealed acute macrocytic anemia (hemoglobin concentration, 7.9 g/dl; mean corpuscular volume, 108.1 fl; and mean corpuscular hemoglobin, 35.3 pg; Table 1(a)). A detailed examination was performed to determine the reason for these results (Table 1(b)). Hemolysis, elevated liver enzymes, low platelet count (HELLP) syndrome; hemolytic uremic syndrome (HUS); and thrombotic thrombocytopenic purpura (TTP) were unlikely. Systemic lupus erythematosus is reported as a disease that causes anemia [7] but was also unlikely because a test for anti-nuclear antibody was negative. Her C3, C4, and erythrocyte-binding IgG, IgA, and IgM levels were also normal. We then suspected the presence of hemolytic anemia and performed several additional examinations.

As shown in Table 1, an increase of reticulocyte and lactate dehydrogenase (LDH) and a marked decrease of haptoglobin (<10 mg/dl) were found. We further examined the LDH fractions and found that LDH1 and LDH2 were markedly increased. Her urine was negative for hemoglobin. These results strongly suggested the presence of hemolysis. In addition, the direct antiglobulin test (DAT) was positive for anti-IgG and negative for anti-C3d. The indirect antiglobulin test was negative. There was no corresponding medical history or symptoms of infection that could have contributed to the observed hemolytic anemia. A blood test for cold agglutinins was negative. Hill et al. have reported that they can diagnose AIHA when there is evidence of hemolytic anemia, the DAT is positive for IgG, and there is no evidence of an alternative cause of hemolytic anemia when the DAT is positive [8]. Accordingly, warm AIHA was diagnosed as the cause of anemia in this case.

**Figure 1:**
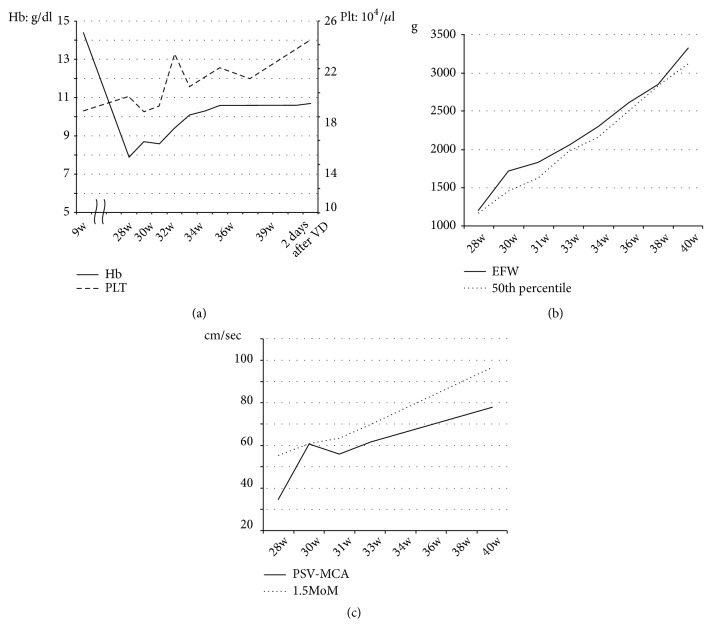
Clinical data during pregnancy and delivery. (a) Transition of hemoglobin concentration and platelet count from an early stage of pregnancy to delivery. Solid line, hemoglobin; dotted line, platelets. (b) Transition of fetal estimated weight (EFW). Solid line, EFW; dotted line, 50th percentile. (c) Transition of middle cerebral artery peak systolic velocity (MCA-PSV). Solid line, PSV-MCA; dotted line; 1.5 MoM.

Maternal blood was regularly tested. We had started iron preparation from onset of anemia empirically, and her hemoglobin level recovered to 10.1 g/dl by 31 weeks of gestation (Figure 1(a)). Although her iron level in the blood was normal, we assumed that iron deficiency might have coexisted and kept iron supplementation. The DAT remained positive at 30 and 34 weeks of gestation. Fetal estimated weight and middle cerebral artery peak systolic velocity (MCA-PSV) were assessed every 2 weeks via ultrasound examination to monitor effects of the anemia (Figures 1(b) and 1(c)), and these factors remained in the normal range.

Labor started spontaneously at 40+1 weeks of gestation, and a normal female newborn was delivered. Her Apgar score was 9/9 (1/5 min), and her body weight was 3575 g. The total bleeding amount was 330 g, and the duration of labor was 380 minutes. No notable event occurred during delivery or the postpartum period. On the days following delivery, the patient's hemoglobin concentration was 10.7 g/dl. The neonatal hemoglobin concentration was 13.6 g/dl. At 2 days of age, the newborn was treated with 24-hour phototherapy because of neonatal jaundice. Both the mother and neonate were discharged on postdelivery day 5.

After discharge, the patient's DAT and hemoglobin concentrations were regularly assessed on an outpatient basis. Her DAT remained positive at 32, 95, and 203 days after delivery. Her hemoglobin level and blood platelet count were normal at 100 days after delivery. From approximately 150 days after delivery, the patient frequently observed nose bleeding and subcutaneous hemorrhage. A blood test at 203 days revealed an extremely low platelet count at 8000/*μ*l (Table 2). The patient was admitted to the Department of Hematology, and bone marrow aspiration was performed. The form of megakaryocytes was normal, and no malignant cells were detected. A diagnosis of ITP was made. Because it occurred after the development of AIHA, Evans syndrome was considered.

**Figure 2:**
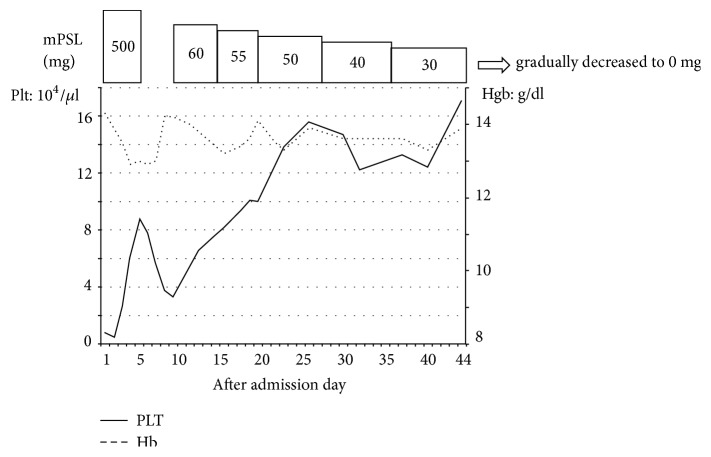
Transition of hemoglobin concentration and platelet count after development of idiopathic thrombocytopenic purpura (ITP) and subsequent treatment with corticosteroids. Solid line, platelets; dotted line, hemoglobin. The treatment procedure is also shown.

Treatment with corticosteroids was initiated (3 days of methylprednisolone 500 mg) on the day after hospitalization, and the patient's platelet count recovered to 88,000/*μ*l. Notably, after completion of the corticosteroids treatment, the platelet count decreased again, and oral administration of prednisolone 60 mg was initiated. Progress was satisfactory, and the prednisolone dosage was gradually decreased to 0 mg (Figure 2).

At 1 year after completing corticosteroid treatment, hemoglobin and platelet counts remained in the normal range.

## 3. Discussion

We presented a pregnancy complicated with Evans syndrome, which was first diagnosed as AIHA during the third trimester of pregnancy. AIHA is occasionally accompanied by ITP (0.8-3.7%), a condition known as Evans syndrome [6]. Thus far, there are few reports that refer to the relationship between Evans syndrome and pregnancy. In most cases of pregnancy complicated with Evans syndrome, anemia and thrombocytopenia occur during pregnancy or shortly after delivery [5, 9]. We could not find any reports of the development of ITP long after delivery. In the present case, we closely followed up and observed the development of ITP at 203 days after delivery. The onset of anemia and thrombocytopenia occurred at extremely different times. Although the mechanism is unclear, it should be considered that ITP can develop long after the onset of AIHA and delivery. Close and long-term follow-up is recommended when AIHA is diagnosed during pregnancy.

The two most common causes of anemia during pregnancy and the puerperium are iron deficiency and acute blood loss. Other causes include inflammation, malignancy, megaloblastic anemia, and acquired hemolytic anemia. In our case, acute blood loss, megaloblastic anemia, and malignant diseases were unlikely. Blood tests showed an increase of reticulocytes and LDH levels and a decrease of the haptoglobin level. As a result, an acquired hemolytic disease was suspected because the patient had no history of congenital hemolytic anemia.

Hemolysis occurs under many conditions, such as HELLP syndrome, acute fatty liver of pregnancy (AFLP), HUS, and TTP [3], or as a result of medication. In our case, the blood test results and medication history did not correspond to HELLP syndrome, AFLP, HUS, TTP, or drug-induced hemolysis. Furthermore, the DAT for anti-IgG was positive, indicating the likelihood of AIHA according to a diagnostic approach shown in a previous review [8]. AIHA is a disease characterized by the development of anti-erythrocyte autoantibodies and the destruction of erythrocytes. This disease is classified as warm (65%), cold (30%), and mixed (5%) type [10]. The main clinical features of AIHA are acute anemia, hemolysis, and a positive DAT result.

The presence of RBC autoantibodies is not consistently associated with hemolytic anemia. Silent RBC autoantibodies have been detected in healthy blood donors, pregnant women, and patients with autoimmune disorders. Among 60 cases of silent AIHA, five cases occurred in pregnant women, and this disease had no effect on the course of pregnancy, fetal development, or health of the newborns [11]. Hoppe et al. reported that autoimmunization against RBCs increases during pregnancy [12]. Issaragrisil et al. reported 14 cases of pregnancy-associated AIHA, with 10 of the 14 cases being women who became pregnant during the AIHA remission period, and the AIHA worsened during pregnancy [7].

In the present case, the detection of silent AIHA was uncertain, but early in pregnancy, the blood count was normal, and anemia occurred at 28 weeks of gestation. Although the exact mechanism remains unknown, this report and previous reports suggest that AIHA develops or worsens during pregnancy. Wikman et al. suggest that cytokines such as interleukin-8 may reflect antibody activation [13]. Cytokine activity markedly changes during pregnancy, and thus, pregnancy may induce antibody reactions. Further studies are required to reveal the mechanism behind the relationship between AIHA and pregnancy.

Issaragrisil et al. reported several cases of AIHA in pregnant women and noted that, in most cases, patients require treatment with corticosteroids or termination [7]. Lauzikiene et al. reported a case of resistance to corticosteroid treatment that required termination [3]. In our case, the hemoglobin level recovered and did not require corticosteroid treatment.

As warm autoantibody is an IgG antibody, it passes through the placenta and may cause fetal hemolytic anemia. Chaplin et al. reported four stillbirths and one neonatal death among 19 cases reviewed [14]. Lawe et al. reported a case that required four exchange transfusions for neonatal hyperbilirubinemia [15]. In the present case, we performed repeated ultrasonography to evaluate fetal growth and MCA-PSV, and we carefully followed up the fetal condition. The pregnancy and delivery were completed uneventfully, and the patient and her baby were uneventfully discharged from the hospital.

In conclusion, we experienced a case of Evans syndrome diagnosed from acute anemia at 28 weeks of pregnancy. In the case of acute anemia during pregnancy, a thorough investigation of the cause is important. When AIHA is diagnosed during pregnancy, close and careful observation is essential because it can worsen both the fetal and the maternal condition. In addition, close follow-up via repeated blood tests after delivery is recommended because it enables an early diagnosis of ITP.

## Figures and Tables

**Table:** (a) Total blood count and urine test data when anemia developed at 28 weeks of gestation

WBC	8.2 x10^3^	(4.0-9.0 x10^3^)	/ul

RBC	2.73 x10^6^	(3.80-4.80 x 10^6^)	/ul

Hb	7.9	(11.0-15.0)	g/dl

PLT	19.7 x 10^4^	(12-35 x 10^4^)	/ul

MCV	108.1	(83.0-99.0)	fl

MCH	35.3	(28.4-34.6)	pg

Reticulocyte	20 x 10^4^	(3-10 x 10^4^)	/ul

	73.4	(5.0-20.0)	‰

Urine hemoglobin	negative		

**Table:** (b) Detailed blood test results at 29 weeks of gestation

AST	17	(8-30)	IU/l

ALT	10	(5-35)	IU/l

Creatinine	0.51	(0.30-0.90)	mg/dl

LDH	312	(106-211)	IU/l

LDH 1	40	(21-31)	%

LDH 2	37	(28-35)	%

LDH 3	15	(21-26)	%

LDH 4	4	(7-14)	%

LDH 5	4	(5-13)	%

UA	4.3	(2.5-7.0)	mg/dl

Total Bilirubin	0.8	(0.2-1.0)	mg/dl

C3	100	(65-135)	mg/dl

C4	29	(13-35)	mg/dl

Antinuclear antibody	negative		

Fe	100	(55-180)	ug/dl

Ferritin	40.3	(5-152)	ng/ml

UIBC	290	(130-320)	ug/dl

Haptoglobin	10>		mg/dl

Direct Coombs	positive		

Indirect Coombs	negative		

IgG	757	(870-1700)	mg/dl

IgA	160	(100-410)	mg/dl

IgM	70	(35-220)	mg/dl

Cold agglutinin reaction	128	(<256)	titer

**Table 2:** Total blood count data when thrombocytopenia developed at 203 days after delivery (reference ranges are shown).

WBC	5.4 x10^3^	(4.0-9.0 x10^3^)	/ul

Hb	14.3	(11.0-15.0)	g/dl

PLT	0.8 x10^4^	(12-35 x10^4^)	/ul

MCV	82.4	(83.0-99.0)	fl

MCH	28.0	(28.4-34.6)	pg

Direct Coombs	positive		

## References

[1] Ronnenberg A. G., Wood R. J., Wang X. (2004). Preconception hemoglobin and ferritin concentrations are associated with pregnancy outcome in a prospective cohort of Chinese women. *Journal of Nutrition*.

[2] Mishra A., Dave N., Viradiya K. (2013). Fatal anaphylactic reaction to iron sucrose in pregnancy. *Indian Journal of Pharmacology*.

[3] Laužikiene D., Ramašauskaite D., Luža T., Lenkutiene R. (2015). Pregnancy Induced Autoimmune Warm Antibodies Hemolytic Anemia: A Case Report. *Geburtshilfe und Frauenheilkunde*.

[4] Garvey B. (2008). Rituximab in the treatment of autoimmune haematological disorders. *British Journal of Haematology*.

[5] Phupong V., Sareepapong W., Witoonpanich P. (2004). Evans syndrome and pregnancy: A case report. *BJOG: An International Journal of Obstetrics & Gynaecology*.

[6] Jaime-Pérez J. C., Guerra-Leal L. N., López-Razo O. N., Méndez-Ramírez N., Gómez-Almaguer D. (2015). Experience with Evans syndrome in an academic referral center. *Revista Brasileira de Hematologia e Hemoterapia*.

[7] Issaragrisil S., Kruatrachue M. (1983). An Association of Pregnancy and Autoimmune Haemolytic Anaemia. *European Journal of Haematology*.

[8] Hill Q. A., Stamps R., Massey E., Grainger J. D., Provan D., Hill A. (2017). The diagnosis and management of primary autoimmune haemolytic anaemia. *British Journal of Haematology*.

[9] Lefkou E., Nelson-Piercy C., Hunt B. J. (2010). Evans' syndrome in pregnancy: A systematic literature review and two new cases. *European Journal of Obstetrics & Gynecology and Reproductive Biology*.

[10] Rai P., Sharma G., Singh D., Garg J. (2017). Rare presentation of mixed autoimmune hemolytic anemia in children: Report of 2 cases. *Journal of Laboratory Physicians*.

[11] Mauro F. R., Trastulli F., Alessandri C. (2017). Clinical relevance of silent red blood cell autoantibodies. *Haematologica*.

[12] Hoppe B., Stibbe W., Bielefeld A., Pruss A., Salama A. (2001). Increased RBC autoantibody production in pregnancy. *Transfusion*.

[13] Wikman A., Axdorph U., Gryfelt G., Gustafsson L., Björkholm M., Lundahl J. (2005). Characterization of red cell autoantibodies in consecutive DAT-positive patients with relation to in vivo haemolysis. *Annals of Hematology*.

[14] Chaplin Jr. H., Cohen R., Bloomberg G., Kaplan H. J., Moore J. A., Dorner I. (1973). Pregnancy and Idiopathic Autoimmune Haemolytic Anaemia: A Prospective Study during 6 Months Gestation and 3 Months Post‐Partum. *British Journal of Haematology*.

[15] Lawe J. E. (1982). Successful exchange transfusion of an infant for AIHA developing late in mother's pregnancy. *Transfusion*.

